# Mr. Ring, on Vaccine Inoculation

**Published:** 1802-02

**Authors:** John Ring

**Affiliations:** New Street, Hanover Square


### *To the Editors of the Medical and Phyfical Journal.*

GENTLEMEN,

FROM the active and decided part which you have taken in favour of Vaccine Inoculation, you cannot be indifferent to its progrefs; I therefore fend the following intelligence for infertion, if you deem it worthy of a place in your ufeful mifcellany.

By a letter received from the Rev. Mr. Pitt, chaplain to the Englifh Factory at Peterfburgh, I am informed, that vaccine virus, tranfmitted by Dr. Rogers, has at length fucceeded in producing the happy preventive of the Small-pox in that city. The practitioner who was fo fortunate as to confer this incalculable benefit on that vaft empire was Dr. Simpfon, who, as Dr. Rogers is informed, inoculated his own children with fuccefs.

Dr. Sacco, of Milan,* has fent to Dr. Jenner fome vaccine matter, originally procured from a cow in Lombardy. With matter from the fame fource, Dr. Sacco had inoculated above eight thoufand perfons, and was in poffeffion of a confiderable number of facts, to prove its efficacy in refifting the Small-pox.

Some of this matter, with which I was favoured by Dr. Jenner, has excited the genuine puftule; and, in my own practice, and in that of others, is now fpreading the vaccine preventive in every direction.

Dr. Woodville, by whom this valuable and interefting prefent from Dr. Sacco was tranfmitted to Dr. Jenner, has alfo been fo fortunate as to produce the genuine Cow-pox with the virus which he himfelf received from Dr. Sacco.

Thefe

* See Retrofpect.

Thefe are the firft inftances of the production of the vaccine puftule in England, by foreign matter. Such an event cannot but be a frefh caufe of exultation to every benevolent mind, and particularly to thofe who have exerted themfelves in promoting the new practice; as it will ftimulate other medical men, in other countries, to feek for indigenous matter, and accelerate the propagation of vaccinifm over the whole face of the globe.

It muft afford additional fatisfaction to your readers to learn, that the Cifalpine Government has refolved to render the new inoculation general, throughout all the territories of the Republic.

I lately received a letter from Dr. De Carro, the founder of Vaccine Inoculation on the Continent of Europe, in which he fays, " You know I had the happinefs to be the firft phyfician on the continent who adopted the new method, and that my own fons were the fubjects whom I took for that experiment. My example was foon followed by innumerable practitioners; and now it would be abfolutely impoffible to name the countries and towns that have adopted it. It would be fhorter, and more accurate to fay, *all Europe*. After very nearly three years of fuccefs, I need not tell you what I think of vaccination: I know of no encomium that can give an adequate idea of that bleffing to mankind.

" Among the extravagant publications againft vaccination, I have lately read one, in which the author, a phyfician at Frankfort, ferioufly endeavours to prove, by prophecies of the Holy Scriptures, and of fome fathers of the church, that it is no lefs than Antichrift!!!—Nothing reflects greater honour on the Jennerian Doctrine than fuch attacks; and nothing fhews more, how difficult, or rather impoffible it is, to refute it properly, than the neceffity of having recourfe to fuch pitiful refources.

" Remember me to Dr. Jenner; no medical man ever excited my admiration and veneration fo much; he is not only great by the magnitude of his difcovery, but he is alfo great by the manner in which he conducted his refearches, by the perfection which he gave to them before he publifhed his work, and by the extreme modefty with which he fpeaks of himfelf."

Dr. De Carro† did me the honour to fend me a copy of his own publication, which has had a rapid fale, and of which a fecond edition, with important additions, is in the prefs. With this he has fent another copy, ftating, that it is for one of the beft Englifh Reviewers, who may think it worth while to give an account of it.

He

† See Retrofpect.

He remarks, that as far as he can judge by periodical publications, it does not ſeem that, in England, we are well informed of the immenſe increaſe of foreign literature on the ſubject of Vaccine Inoculation.

Your Journal having eminently diſtinguiſhed itſelf above all others, by giving the moſt faithful and regular account of the riſe, progreſs, and preſent ſtate of the practice, I beg your acceptance of Dr. De Carro's work. It is a tribute due to the ability, candour, and impartiality, which you have diſplayed on this moſt intereſting occaſion.

As you have not relinquiſhed the review of foreign books, I truſt you will deem a critique on the work of ſo celebrated an author, and of ſo liberal and enlightened a man as Dr. De Carro, not unworthy of a place in your valuable publication.

*New Street, Hanover Square,*
*Jan.* 15, 1802.

I am, &c.

JOHN RING.

